# Gummy Smile Prevalence Among Ha’il City Female Young Adults and Its Impact on Quality of Life: A Cross-Sectional Study

**DOI:** 10.7759/cureus.51302

**Published:** 2023-12-29

**Authors:** Arwa A Al Sayed, Bodor Z Alshammari, Abdullah R Alshammari, Mariam B Aldajani, Falah R Alshammari

**Affiliations:** 1 Periodontics and Dental Implant, Sijam Dental Center, Riyadh, SAU; 2 Dentistry, University of Ha'il, Ha'il, SAU; 3 Nursing, Ministry of Health Saudi Arabia, Ha'il, SAU; 4 Paediatric Dentistry, Faculty of Dentistry, King Abdulaziz University, Jeddah, SAU; 5 Dental Public Health, College of Dentistry, University of Ha'il, Ha'il, SAU

**Keywords:** ohip, saudi arabia, young adults, oral health, gummy smile

## Abstract

Background: Gummy smile (GS) has a direct effect on individuals, especially among young adults, because of its association with smile avoidance. The younger populations are sensitive about their smiles and prefer aesthetic, beautiful smiles, a lack of which can negatively impact their quality of life.

Objectives: This study aims to measure the GS prevalence among young adults aged 16 to 18 attending high schools in Ha’il City, Saudi Arabia, evaluating oral health related to quality of life (OHQoL) in those suffering GS by using the OHQoL questionnaire (OHIP-14).

Methods: A cross-sectional study was conducted on 385 female high school students located in Ha’il. Students with GS took a survey on oral health using OHIP-14. For this, SPSS was used to analyze the data.

Results: The study included 200 people with GS (52%). The mean age was 18±0.01. The prevalence of GS was analyzed, with a mean value of 4.68±1.2 mm, indicating most students had GS ranging between 4 and 5 mm. The most frequent value for all items in the OHIP-14 questionnaire was 1, indicating that students often had their quality of life affected. The non-parametric Kruskal-Wallis test indicated the results had a significant value (p < 0.05), showing a positive and significant association.

Conclusion: Based on the OHIP-14 questionnaire and respecting the methodology, it was concluded that the quality of life has been affected for all female students with GS. The high prevalence for ages 16-18 showed most students agreed their lives were being affected by GS and their condition needed to be treated. It was also confirmed by the significant association of GS with items of oral health and quality of life.

## Introduction

Smiles with excessive exposure of the gum (more than 3 mm) are known as gummy smiles (GS). GS has a direct effect on individuals, especially young adults because it is associated with smile avoidance [1,2]. Thus, it affects their quality of life [3]. It constitutes a widespread condition that occurs in 10.5-29% of young adults, with a higher frequency in women [4]. In Saudi Arabia, the study was conducted among laypeople and a dentist, among whom 66.2% of respondents agreed to GS treatment related to aesthetics to improve their quality of life. Compared to older people, the younger population did not accept the GS, as, according to them, it was spoiling their attractiveness and aesthetics [5].

Having a good smile has been linked to having a good shape of the dental and jaw outline, with associations with the function of the lip muscles [6,7]. According to Camara, a fine-drawn smile in an individual is achieved when there is an arc curve of gums at the edge of the incisal edge of the central, lateral, canines, and premolars with less than 3 mm of gum exposure [8]. Levi et al. [3] argued that, although GS can appear at any age among people, young people are the most affected because of the aesthetic aspect, as it affects their ability to smile and socialize. The World Health Organization defined oral health as “oral health that enables an individual to speak, eat and socialize without active disease, discomfort or embarrassment” [9].

Clearly, an understanding of the prevalence of GS is an important starting point when trying to identify its effect on young adults. It can also help in outlining treatment strategies to improve young people’s oral health. GS is related to the aesthetic aspect among young adults because it is correlated with their smile and social life, affecting their self-confidence. If there is a need to take action to treat it, it is important to seek suggestions regarding appropriate treatment possibilities later on and look for factors related to GS [1-3]. This study aimed to measure the GS prevalence among female young adults aged 16 to 18 attending high schools in Ha’il City, Saudi Arabia, evaluating oral health related to the quality of life (OHQoL) of those suffering from GS by using the OHQoL questionnaire (OHIP-14).

## Materials and methods

Study design

This study utilized a cross-sectional design. It was conducted among 385 young females aged 16 to 18 attending high schools in Ha’il, Saudi Arabia. The participants were recruited through random sampling by selecting females from high school settings throughout the city. Ethical approval was achieved through the University of Ha’il (H-2023-407).

Study area

This study was conducted in Ha'il, Saudi Arabia.

Study population

Female participants from high schools in Ha’il City, Saudi Arabia, were included in the study.

Inclusion and exclusion criteria

The inclusion criteria for the study on evaluating the prevalence of gummy smiles and their relation to quality of life included Saudi females at the high school level. The exclusion criteria include all females less than 16 and greater than 18 years old. It excluded females without gummy smiles or smiles with less than 2 mm gingival display. Furthermore, the study also excluded all high school male students.

Sample population

The sample size was calculated using an online tool, taking into account a 5% sampling error and a 95% confidence level, after taking the student numbers from the Ministry of Education.

The sample size of this study is 385, including all females who are eligible for inclusion criteria. We have calculated our sample size using standard online tools using the following formula:

N=(Zα)2 × [p(1-p)]/d2

where n is the estimated sample size; Zα at 5% level of significance is 1.96; d is the level of precision and is estimated to be 0.05; p is the proportion of previous studies (0.5); actual sample size = primary sample size × design effect (estimated to be 1.5).

The expected response rate is estimated to be 80%.

Z = 1.96, p = 0.5, d = 0.05

n = (1.96)2×([0.5 * (1 - 0.5)]/ 0.052)

n = 0.9604/0.0025 = 384.16

n ≈ 385

The sample size is equal to 385.

Recruitment process

In this research, two phases have been used to collect data. For the first phase, we selected the high schools from which we would recruit female students. The second phase included participants (female students who met the inclusion criteria) from the selected schools.

As there were many female high schools in Hail City, the authors decided to select schools randomly from each area within Hail City. So, two schools were selected from each direction (north, west, east, and south).

The school manager provided the student schoolbook number, and based on that, female students were randomly selected from each grade (class within the school) in the selected schools.

Data collection

After an intensive literature review and expert consultation, the questionnaire was made. It consists of all the relevant information regarding our research goals and objectives. Part one consisted of demographic data, including age, education level, measurement of a gummy smile, etc. Part 2 contained an OHIP-14 questionnaire regarding gummy smiles and quality of life. The responses were collected through an offline questionnaire. All eligible participants in the schools received copies of the final questionnaire. We filled out the questionnaire until we achieved the desired sample size. Furthermore, to evaluate the prevalence of gummy smiles in relevant studies, an electronic search was made through PubMed, Scopus, etc. All possible searches were made, mentioning all possible combinations and checking for duplications.

Calibration

For calibration, the GS diagnosis took place in the dental clinic of the examiners until agreement was reached. Two individual examiners (BD and MR) ran the GS investigation inside a private room within the school. GS was measured using a dental probe (Williams probe). The distance between the tip of the lower margin of the upper lip and the gingival margin of the central incisor was measured after the participants signed the consent form.

Data analysis

SPSS (version 25) was utilized to calculate the descriptive analysis for the study, including frequency and percentage. Part 2 questions were analyzed with intra- and inter-examiner agreement, considering kappa values of 0.83 and 0.82, respectively. OHIP-14 was adapted to determine those suffering from GS. Because the total OHIP-14 response was 28, the order impact of GS was classified as 0 if there was no impact, as low impact if 1 ≤ OHIP-14 ≤ 9, as medium if 9 < OHIP-14 ≤ 18, and as strong if 18 < OHIP-14. Apart from a descriptive study, this study used item-total correlation considering the alpha values. Although the Kruskal-Wallis test was used when there was no normal distribution, a p-value of 0.05 was used to test the significance of the different variables.

## Results

In the current cross-sectional study, the high school students of Ha’il City, Saudi Arabia, participated. A total of 385 students were interviewed for gummy smiles, including their demographics such as age, education, inheritance, and ongoing treatment. Out of 385, 52% had a gummy smile. The age range was 16-18, and the mean age was 18±0.01. The prevalence of gummy smiles was analyzed with a mean value of 4.68±1.2 mm, indicating that most students had gummy smiles ranging between 4 mm and 5 mm. Most students agreed that their gummy smile is an inherited trait and their treatment is going on as shown in Table 1.

**Table 1:** Descriptive data of high school students with gummy smiles.

Variables	Mean	Count (%)
Age		
16	18±0.01	53 (26.5)
17		95 (47.5)
18		52 (26)
Education level		
High school		200
Gummy smile (mm)		
2-4	4.68±1.2	87 (43.5)
5-7		112 (56)
8-10		1 (0.5)
Inheritance		
No		76 (38)
Yes		124 (62)
Treatment		
No		82 (41)
Yes		118 (59)

The questionnaire with OHIP-14 items was developed to analyze the quality of life related to gummy smiles among high school students in Saudi Arabia, as shown in Table 2. The Likert scale with different options was used to assess its prevalence with more or less frequency. The most frequent value for all items was 1, which indicated that quality of life was affected very often among students. A gummy smile affected 80% of students in pronouncing the words and 60% for worsening taste. The highest percentage for the effect of a gummy smile was 86% and 85% for feeling tense and feeling life in general less satisfying, respectively. All of the items indicated the students with gummy smiles had disturbed their quality of life as they felt less comfortable while talking, which damaged their confidence level.

**Table 2:** Prevalence of students with gummy smiles.

	Very often (n)	Fairly often (n)	Occasionally (n)	Hardly ever (n)	Never (n)	Don’t know (n)	Mode	Decision
Trouble pronouncing any words	160	8	12	10	4	6	1	Very often
Sense of taste has worsened	120	47	8	6	5	14	1	Very often
Had painful aching in your mouth?	110	40	14	22	10	4	1	Very often
Uncomfortable to eat any food	140	33	5	8	8	6	1	Very often
Feel self-conscious	148	24	8	8	6	6	1	Very often
Feel tense	170	14	4	4	6	2	1	Very often
Unsatisfactory diet	162	16	10	6	2	4	1	Very often
Totally unable to function	172	12	8	4	4	0	1	Very often
Had to interrupt meal	158	20	12	2	6	2	1	Very often
Found it difficult to relax	136	36	18	6	2	2	1	Very often
Been a bit embarrassed	130	40	16	12	2	0	1	Very often
Been a bit irritable with other people	152	18	12	8	6	4	1	Very often
Had difficulty doing your usual jobs	126	38	14	10	10	2	1	Very often
Felt that life in general was less satisfying	150	22	16	6	4	2	1	Very often

**Figure 1:**
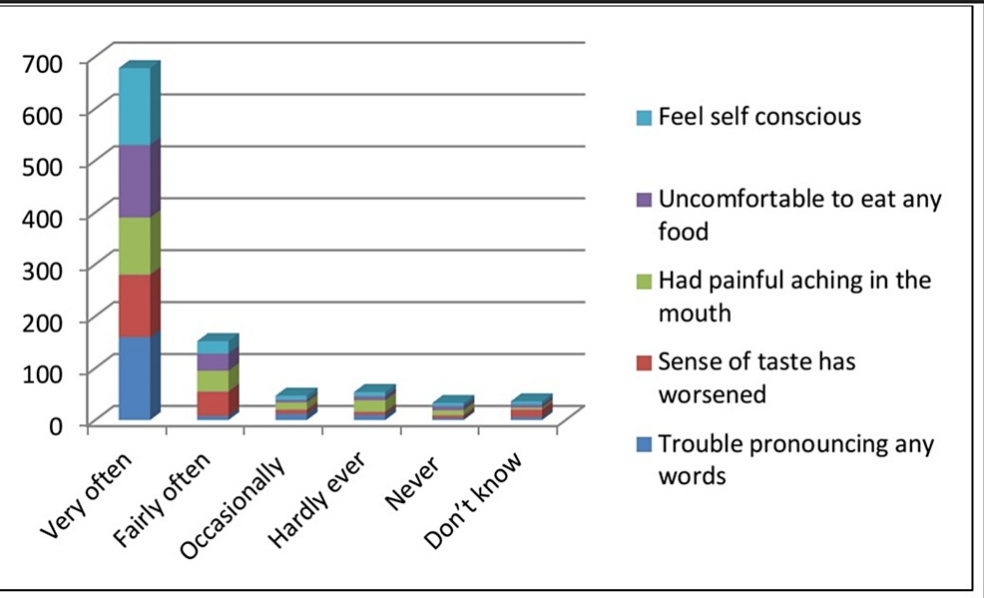
Prevalence for effect of quality of life among gummy students.

Figure 1 shows the prevalence of quality of life being affected by a gummy smile. The results showed that the majority of students with gummy smiles agreed very often with the effects on oral health that they feel self-conscious, uncomfortable eating food, and many more effects.

The items for OHIP-14 were analyzed for correlation and reliability, as shown in Table 3. The correlation of the total population with the gummy smile students was compared, considering the kappa values of 0.82 and 0.83. In the total sample population, Cronbach’s alpha coefficient for the total OHIP-14 items was 0.942, and item total correlation ranged from 0.558 to 0.879. All the values showed reliability above 70%, while the most efficient was 87%, except for one item with a reliability of 53%, indicating that it did not correlate with other items in the scale. Item correlation for students with gummy smiles also showed better reliability for each individual item except the one with 56%, which did not correlate well with others.

**Table 3:** Item total correlation, and alpha if item deleted for individual OHIP-14 items. α = alpha.

	Total sample		Students with gummy smile	
Items	Item-total correlation	α if item deleted	Item-total correlation	α if item deleted
Trouble pronouncing any words	0.734	0.952	0.668	0.937
Sense of taste has worsened	0.761	0.951	0.811	0.933
Had painful aching in your mouth?	0.558	0.956	0.568	0.942
Uncomfortable to eat any food	0.833	0.949	0.822	0.932
Feel self conscious	0.849	0.949	0.821	0.932
Feel tense	0.741	0.952	0.661	0.938
Unsatisfactory diet	0.786	0.951	0.752	0.934
Diet been unsatisfactory	0.754	0.951	0.742	0.935
Had to interrupt meal	0.879	0.948	0.722	0.936
Found it difficult to relax	0.829	0.949	0.874	0.930
Been a bit embarrassed	0.770	0.951	0.812	0.924
Been a bit irritable with other people	0.640	0.954	0.731	0.935
Had difficulty doing your usual jobs	0.816	0.950	0.661	0.940
Felt that life in general was less satisfying	0.679	0.953	0.797	0.933

The test for the effect of gummy smiles on oral health and quality of life among high school students was conducted as shown in Table 4. The non-parametric Kruskal-Wallis test indicated a significant difference in gummy smiles for quality of life. All the questions tested for affecting the oral health of students indicated a significant value (p<0.05). The significant results showed a positive relationship, which means students with gummy smiles have affected oral health and quality of life.

**Table 4:** Effect of gummy smile on the quality of life of students with gummy smile. Grouping variable: gummy smile.

	Trouble pronouncing any words	Have you felt that your sense of taste has worsened?	Have you found it uncomfortable to eat any food?	Have you felt self-conscious?	Have you felt tense?	Has your diet been unsatisfactory?	Have you had to interrupt a meal?
Median	1.00	1.00	1.00	1.00	1.00	1.00	1.00
Chi-Square	8.185	8.459	8.570	5.656	6.849	9.994	10.156
df	2	2	2	2	2	2	2
Asymp Sig.	0.017	0.015	0.014	0.059	0.033	0.007	0.006

## Discussion

This school-based study conducted in Ha’il aimed to measure GS prevalence among young adults aged 16 to 18 years old and evaluate OHQoL with those suffering from GS by using the OHQoL questionnaire (OHIP-14). In this cross-sectional study, a total of 385 students were interviewed for GS. The mean age was 18±0.01. The GS prevalence was analyzed, finding a mean value of 4.68±1.2 mm, which indicates that most students with GS ranged between 4 and 5 mm. The OHIP-14 questionnaire showed 1 as the most frequent value for all items, which indicated that quality of life was often affected among students. GS had affected 80% of students in regard to pronouncing words and 60% regarding the worsening of taste. The highest percentage prevailed for the effect of GS, which was 86% and 85% for feeling tense and feeling life in general was less satisfying, respectively. In the total sample population, Cronbach’s alpha coefficient for the total OHIP-14 items was 0.942, and the total correlation ranged from 0.558 to 0.879. All the values showed a reliability of above 70%, whereas the most efficient was 87%, and one item had 53% reliability, indicating it did not correlate with the other items in the scale. The non-parametric Kruskal-Wallis test indicated a significant value (p < 0.05), which showed a positive and significant association.

Although our study showed the prevalence of GS in female students only, research conducted by Tjan and Miller [10], Dong et al. [11], and Rigsbee et al. [12] demonstrated that between 10.58% and 29.9% of younger people experience gingival display (GD), with greater prevalence in women [6,11,12]. Moreover, 53 (94.6%) of the 56 patients had GD when they smiled, whereas only 3 (5.4%) showed no GD at all. Overall, 47 (88.7%) of the 53 GD patients were female.

As per our research, 56% of the students had a 5-7 mm GS. According to research by Peck et al., of the 53 individuals who had GD when they smiled, 40 (75.5%) showed GD ≥ 4 mm, and 13 (24.5%) showed GD < 4 mm. Upper lip movement from rest to maximum smile in a reference group (n = 88) of North American Whites has been reported to be 5.2±1.6 mm, indicating that, under a normal distribution, only 4% of persons would present with >8 mm of lip movement. In patients with prominent gingival smiles, the upper lip movement was reported to be 6.2±1.8 mm [13]. Under a normal distribution, an upper lip movement of >8 mm would be expected in <16% of patients [14].

Age influences gingival display. The amount of gingiva displayed is inversely proportional to increasing age. Therefore, a young person will display more gingiva, whereas an older individual will show less. Current study results showed that the prevalence of GS was higher among 16-18-year-old students. According to one study, adolescents between the ages of 15 and 16 were chosen from among all the students enrolled in Bauru’s secondary schools. Of these, those with class II malocclusion reported the highest frequency of CSIs (condition-specific impacts) (54.6%), whereas those with normal occlusion reported the lowest rate (32.7%). The condition had an impact on their daily performance, with eating and smiling being commonly affected activities, while sleeping remained unaffected [15]. A further investigation by Al-Habahbeh et al. [16] found there were notable gender variations in the maxillary anterior area gingival displays during smiling, with women showing more gingivae than men [17].

Gingival broadening, which causes GS, can be brought on by excessive collagen synthesis, fluid retention (edema), or, in rare instances, a rise in the host cell population. Hepatocyte growth factor is an uncommon illness. It has an equivalent impact on men and women at a 1:175,000 phenotypic frequency [18]. Although recessive variations have also been reported, autosomal dominant inheritance is likely to be the mode of transmission [19]. The majority of students (62%) felt GS can be transmitted through genes, according to the results of our study. The study results contrast with those of Al-Habahbeh et al. [16], who showed that 48% of patients with GS had a familial etiology and 52% had GS without a familial etiology, which indicates heredity is not a significant etiological factor for GS [17]. Irregularities in the position of the teeth and jaws have a significant impact on the attractiveness and aesthetics of a smile and one’s quality of life. These irregularities can disrupt social interaction, interpersonal relationships, and mental well-being and may lead to feelings of inferiority [20]. Most orthodontic patients are children and adolescents [21].

It is believed that youth who suffer from uneven mouths have less attractive faces. Such youth can experience negative effects like taunts, nicknames, and other treatments [22]. According to the results of our study, the majority of students (80%, 85%, 86%, etc.) had poor oral health, which was indicative of a lack of self-worth, confidence, and self-consciousness. According to 44% of parents in another article [23], children are teased because of their teeth. Compared to parents of children with less overjet, parents of children with overjet ≥7 mm are 5.5 times more likely to report that their child has been tested. The same study lists 73% of respondents having advice from dentists, 46% having a good facial appearance, 16% having a good speech, and 85% having a good tooth appearance as additional factors contributing to interest in orthodontic treatment. According to recent research, abnormalities in the positions of the jaws and teeth can have negative effects on one’s quality of life at the physical, mental, and social levels [24].

A collection of genetic conditions known as amelogenesis imperfecta (AI) mainly affects the amount and quality of amelogenesis both in primary and permanent dentitions. AI is brought on by mutations in genes that regulate amelogenesis, such as amelogenin, and which are inherited via X-linked, autosomal-recessive, or autosomal-dominant modes of transmission [25]. In a case study, a 17-year-old girl in Iran was referred to the Department of Prosthodontics of Shiraz Dentistry School for treatment because of her unpleasant appearance and dysfunction. Her dental characteristics were similar to those of a hypoplastic-hypomature type of AI. When smiling, her high lip line showed about 6 mm of the cervical gingival tissues (gummy smile) [26].

In Saudi Arabia, although work has been done to enhance understanding of GS among dentists and laypeople, not enough studies have been conducted analyzing GS prevalence in students. This study considered the need to determine the prevalence of GS among high school students and analyzed its effects on oral health and quality of life. For this, the OHIP-14 item scale questionnaire aided in studying the factors affecting the quality of life with regard to GS.

This study is based on a cross-sectional design that is a snapshot study for one time. It required a larger sample size to analyze the effects on their lives and the effects caused by environmental and social factors. The study did not include the female students who did not have gummy smiles as a control to compare with and the study did not compare the three levels of the gummy smile to see whether an increased gummy smile would affect the quality of life more. Furthermore, there is a need to investigate the prevalence among females older than 18 and males in high school and the types of treatment. In addition to treatment for gummy smiles, building confidence and promoting positive self-talk with more confidence is recommended for individuals to progress in their lives.

## Conclusions

Based on the OHIP-14 questionnaire and the methodology, it was concluded that GS affects the quality of life for female students with GS. The high prevalence for ages 16-18 showed most students agreed their lives were being affected by GS and needed treatment. This was also confirmed by the significant association of GS with items of oral health and quality of life. There is thus a need for further study of the effects students face and the best treatments. In addition to this, their self-esteem and confidence could be increased through awareness.
